# Supplementary material for: ATAD2 controls chromatin-bound HIRA turnover
Source: Life Sci Alliance. 2021 Sep 27;4(12):e202101151. doi: 10.26508/lsa.202101151 (PMC8500222; doi:10.26508/lsa.202101151)
Supplement: Supplementary file 2 [file LSA-2021-01151_SdataF3.pdf]

Raw data Western blots Figure 3A

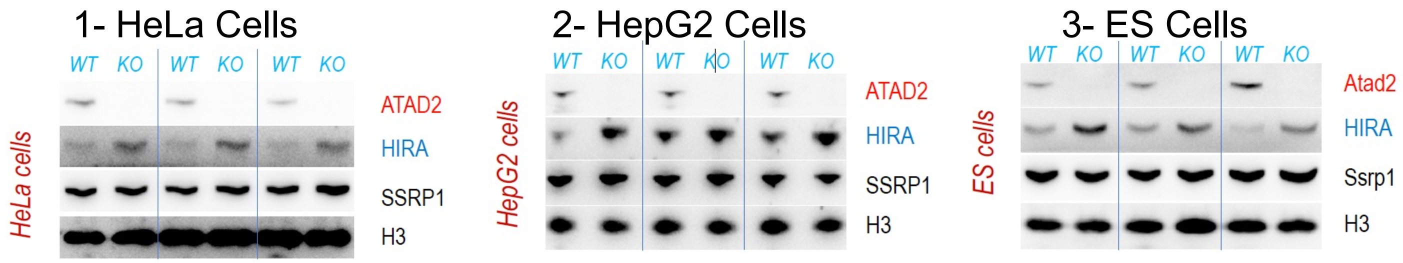

1- HeLa Cells

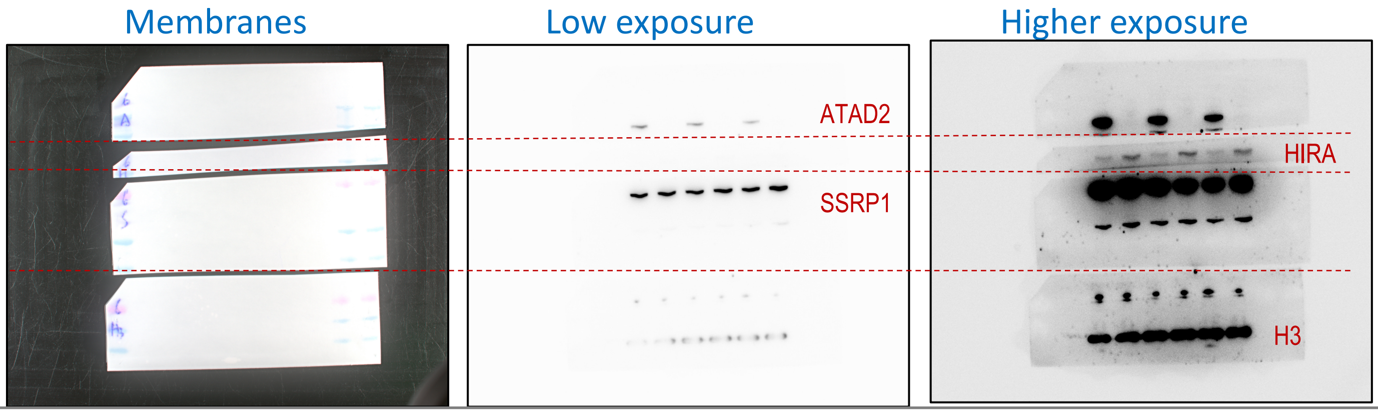

2- HepG2 Cells

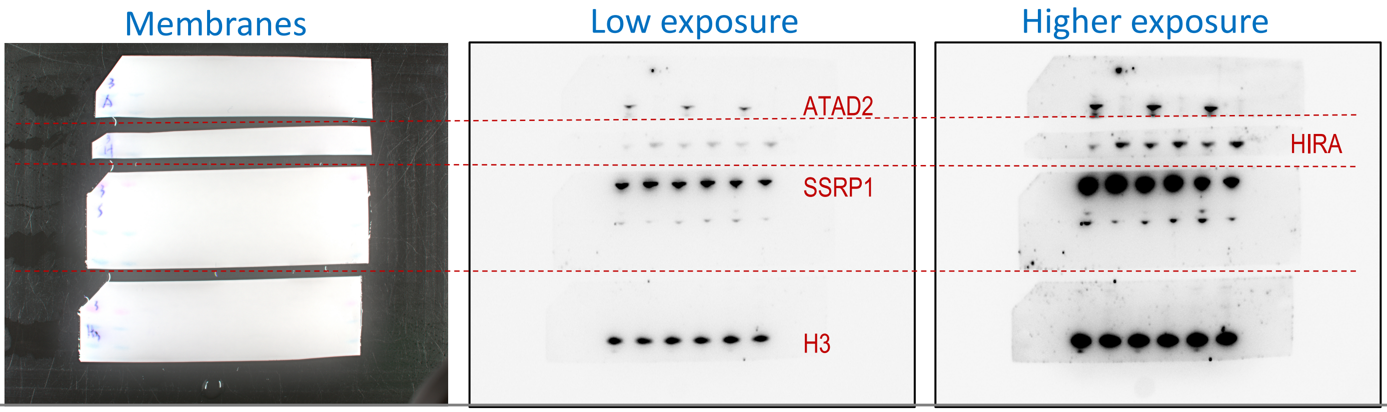

3- ES Cells

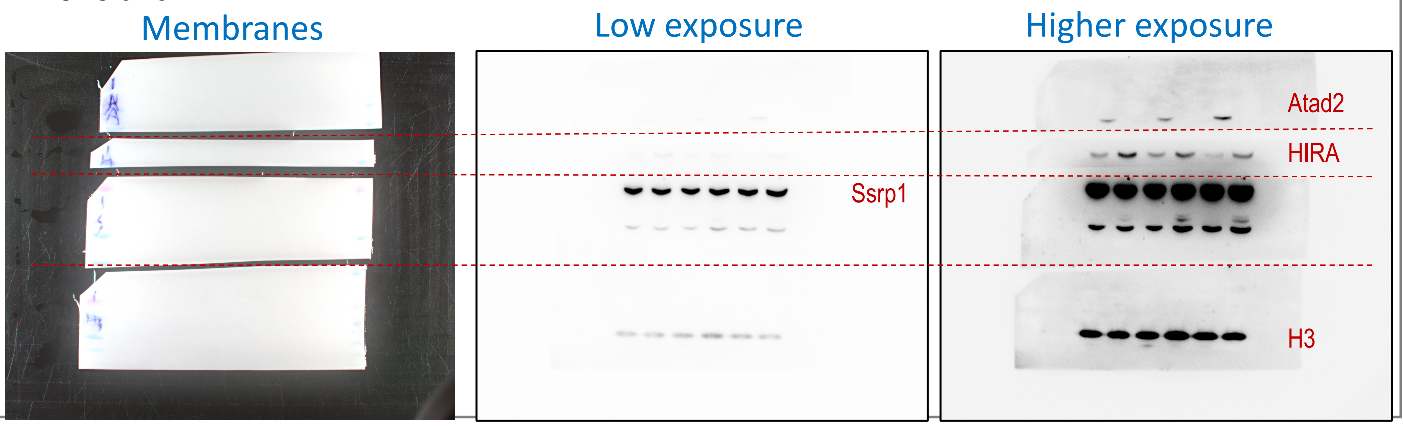

After SDS-PAGE, the blot was cut as shown and each strip was probed with the indicated antibodies.
After revelation, low and high exposures were performed as indicated.
The final Figure was made by choosing the appropriate exposure for each antibody.
The exposures chosen for the final Figure are those labelled by the name of the antibody used.
